# Changes in sleep schedule and chronotype due to COVID-19 restrictions and home office

**DOI:** 10.1007/s11818-020-00277-2

**Published:** 2020-11-17

**Authors:** Naomi Staller, Christoph Randler

**Affiliations:** Eberhard Karls Universität Tübingen, Tübingen, Germany

**Keywords:** Circadian preference, Morningness–eveningness stability scale improved, New ways of working, Social jetlag, Distant learning, Circadiane Präferenz, „Morningness-Eveningness-Stability-Scale improved“, „New ways of working“, Social Jetlag, Distanzlernen

## Abstract

**Background and objective:**

In this study, we researched the effects of the COVID-19 restriction measures on the sleep health of *N* = 681 German residents (mean age: 28.63 years, SD: 10.49 years).

**Methods:**

The data were collected with an anonymous online survey composed of validated questionnaires and additional questions to quantify changed circumstances during the pandemic. Data were collected from May 18 to June 17, 2020, while governmental restrictions were imposed in Germany. We exclusively analysed participants working in home office during this time.

**Results:**

Participants woke up about 1 hour later during the COVID-19 restriction phase, while going to bed at almost the same time as before. During the week, participants slept about an hour longer, while sleep at weekends did not differ significantly. Social jetlag decreased from 1:39 ± 1:00 to 0:49 ± 0:42 min in our sample. The number of children in the household was a significant factor predicting sleep timing. Participants with children living in the same household slept longer and sleep onset was later.

**Conclusion:**

In terms of sleep behaviour and, consequently, sleep health, participants benefited from the transition to home office. They were able to adapt their waking and working hours better to their biological rhythm, which reduced social jetlag.

## Abstract (de)

**Hintergrund und Zielsetzung:**

In dieser Arbeit beleuchteten die Autoren die Auswirkungen der COVID-19-Restriktionsmaßnahmen auf die Schlafgesundheit von *n* = 681 in Deutschland lebenden Personen (mittleres Alter: 28,63 Jahre, Standardabweichung, SD: 10,49 Jahre). Es wird explizit auf die Veränderungen des Schlafverhaltens der Probanden eingegangen und Stellung zu gesundheitswirksamen Aspekten bezogen.

**Methoden:**

Die Daten wurden anhand einer anonymisierten Online-Befragung mit mehreren validierten Fragebogen und Zusatzfragen zu den veränderten Schlaf- und Arbeitszeiten erhoben. Die Datenerhebung beschränkte sich auf die Zeit der Phase der COVID-19-Restriktionsmaßnahmen zwischen dem 18. Mai und 17. Juni. Es wurden ausschließlich Personen befragt, die in dieser Zeit im Home-Office arbeiteten.

**Ergebnisse:**

Die Teilnehmer wachten während dieser Phase etwa eine Stunde später auf, gingen jedoch nahezu zur selben Zeit wie vor der Umstellung zu Bett. Unter der Woche schliefen die Teilnehmer ungefähr eine Stunde länger, die Schlaflänge am Wochenende unterschied sich nicht signifikant. Der Social Jetlag verringerte sich in dieser Stichprobe von 1:39 ± 1:00 auf 0:49 ± 0:42 min. Die Anzahl der Kinder im Haushalt war dabei ein erheblicher Einflussfaktor auf die Schlafzeiten: Teilnehmer mit Kindern im Haushalt schliefen länger, und die Einschlafzeit war später.

**Schlussfolgerung:**

In der vorliegenden Untersuchungsstichprobe profitieren die Teilnehmer hinsichtlich ihres Schlafverhaltens, und daraus folgend ihrer Schlafgesundheit, von der Umstellung auf Home-Office. Die Versuchsteilnehmer konnten ihre Wach- und Arbeitszeiten besser ihrem biologischen Rhythmus anpassen, wodurch sich der Social Jetlag verringerte.

## Introduction

The first known case of COVID-19 caused by the novel coronavirus SARS-CoV‑2 occurred in the Chinese city Wuhan at the beginning of December 2019 [1]. It has since caused a global pandemic with dramatic consequences for public health, economy, and social life. In order to limit viral transmission and relieve the healthcare system, governments imposed restrictions on their populations. These differed considerably among countries. In Italy for example, a national health emergency was called out on March 10, 2020. Hereafter, the population was placed in social isolation and the Italian territory was locked down until May 3, 2020, when some restrictions were eased [2]. During this period, people were not allowed to leave the house except to take care of the necessities of life (e.g., buying food or medicine) [2]. Contradictorily, the restrictions in Sweden were comparatively mild and mainly relied on voluntary compliance with the Public Health Agency’s recommendations [3]. Preschools (students under 6 years old) and compulsory schools (students under 15–16 years old) remained open, while other schools and universities implemented online teaching. All business remained open as long as proper distance between people could be ensured [3]. Outdoor activities (e.g., walking in parks) were unrestricted and encouraged by authorities [3]. Considering the case numbers, the restriction measures in Germany were imposed early [4]. Starting in the middle of March 2020 (hereafter: COVID-19 restriction phase), universities, schools, kindergartens, and not-system-relevant businesses (e.g., beauty salons) were closed. Gatherings were banned, meeting people was allowed in groups of two (or two households) only, in public areas as well as on private property, and a mandatory isolation of people who had been exposed to or currently had COVID-19 was resolved [4].

The uncertain situation and the governmental measures resulted in changes in sleep and sleep timing around the globe. Some research groups showed increasing prevalence of sleep problems (insomnia, sleep loss, poor sleep quality) in healthcare workers and the general population [5–9], while others showed an improvement of sleep health. In India, China, and Italy, people slept later and longer [2, 7, 10, 11]. In a US sample, Gao and Scullin [9] showed improved sleep parameters even though the perceived view of participants differed partly. Leone et al. [12] showed a decrease of social jetlag in an Argentinian sample.

Sleep deprivation related to late chronotypes was a major problem before the COVID-19 restriction phase, resulting in a state of social jetlag for evening-oriented people [13]. A person’s circadian timing depends on exogenous timekeepers (e.g., light cycle) and endogenous timekeepers (e.g., suprachiasmatic nucleus [SCN]; social/environmental timekeepers) [14]. One of those influencing timekeepers is social pressure due to work and school timing [15, 16]. To adapt to the given times, evening-oriented types sleep less than morning types during the week, which leads to a greater morning sleepiness and need for sleep [17]. Contrarily, they show later bed-/rise times and longer times in bed at the weekend [17]. The accumulated sleep debt leads to serious illness [13]. The discrepancy between the internal biological clock and the actual sleep timing due to social factors defines the state of social jetlag. With less social pressure following flexible working hours and home office, the circumstances might be health beneficial in terms of living with the circadian rhythm for evening-oriented people. Under normal circumstances, people have a daily routine which includes fixed events throughout the day (waking up, eating, working, social contacts, sports, etc.). With several of those altered due to the current circumstances, a change in sleep timing might follow. For example, a person wakes early in the morning. Later that day the person watches a movie instead of training soccer in the evening due to social isolation/restrictions. The exposure to blue light at night results in suppression of melatonin and lack of fatigue. Consequently, sleep onset is later than usual. This results in either a reduced sleep duration (waking up at the usual time) or a delayed wake up time. While working in home office, a person does not need to wake up for work at the same time as before (e.g., because the way to work is omitted), and hence rises later to get the same amount of sleep. The daily routine and, in turn, the sleep timing shifts.

It is important to pay attention to sleep during the COVID-19 pandemic because sleep plays a major part in sustainable health. Good night sleep (consisting of sleep duration/quality and timing) is essential to build resilience and cope with the primary and secondary effects of disease [8]. We hypothesized that sleep timing and therefore overall sleep in evening-oriented people during the changed circumstances is more in line with their biological needs and thus beneficial to health.

## Methods

### Setting

This study was carried out by the Department of Biology, Eberhard Karls University Tübingen, Tübingen, Germany. Data were collected in accordance with the Declaration of Helsinki for experiments involving humans approved by the Eberhard Karls University’s ethics committee (Faculty for Economics and Social Sciences: nr. A.Z.: A2.5.4-124_kr).

### Data collection

We started our anonymous online survey on May 18, 2020, and continued until June 17, 2020. We therefore collected data during the most restrictive phase in Germany. Participants were informed about the study via an electronic mailing list (employees and students of the Eberhard Karls University Tübingen; >20,000 mails) and postings on different social media platforms (Facebook/Instagram). The recruitment text included an online link to the questionnaire. The survey was hosted on an online platform (SoSciSurvey) to fulfil the European Union’s data privacy rules and took an average of 12 min ± 5 min (standard deviation, SD) to complete. The theoretical background and study goals but not the hypothesis were declared. We explicitly informed about the voluntariness of the participation, the option to stop the data collection at any point without consequences, and that participation would not be remunerated. The recruitment text was available in German only and formal consent was inquired in advance. The total number of evaluable cases amounted to 681.

### Demographic data

Age, sex, household size, number of children in the household, profession, and the option to work in flexitime were asked for. Profession was later dichotomized into student (*N* = 400) or non-student (*N* = 281). 197 participants were male, 484 were female. Mean age was 28.63 years, SD 10.49 years. *N* = 545 participants noted that there were no children in their household, while *N* = 136 reported one or more children. We explicitly asked for the number of children in the household and not the number of own children, because, for example, students may have travelled back home to their parents during the restriction phase and lived with younger siblings. Thus, children in the household is a better measure than own children, because regardless of relationship (own children/siblings/other cases), children in general may have an impact on sleep during the pandemic.

### Questionnaire

The questionnaire used was composed of validated questionnaires concerning chronotype and sleep duration as well as additional questions to quantify changed circumstances during the pandemic. Examined characteristics were chronotype/midpoint of sleep, sleep duration, and COVID-19-induced changes in sleep/work hours.

### Chronotype

The Morningness–Eveningness Stability Scale improved (MESSi [18, 19]) and the corrected midpoint of sleep (MSF corrected) were used as separate measures to determine the chronotype. The MESSi is composed of three subscales: the morning affect subscale (MA), the eveningness subscale (EV), and the distinctness subscale (DI). Five items in a 1–5 Likert-format represent each scale. The MA is concerned with the affective facet of the morningness–eveningness trait (M/E; e.g., alertness in the morning: “How alert do you feel during the first half hour after having awakened in the morning?”), while the EV queries feeling/mood, energy level, and learning capacity in the evening (e.g., “In general, how is your energy level in the evening?”). The DI shows the subjectively felt amplitude of diurnal active phases (e.g., “There are moments during the day where I feel unable to do anything” with response options ranging from “totally” to “not at all”). Higher MA or EV scores represent higher morning and evening orientation, respectively, while higher DI values indicate higher daytime fluctuations. MESSi’s factorial invariance, structure, and reliability have already been confirmed repeatedly in different languages [18, 20–23]. In addition, actigraphy data corroborated the validity of the MESSi [24]. Cronbach’s α in the current study sample was 0.899 for MA, 0.889 for EV, and 0.775 for DI.

### Sleep duration

We asked for bed and wake times during the week and at weekends to assess sleep duration and the midpoint of sleep, both during and before the COVID-19 restriction phase. Furthermore, a correction algorithm [25] was used to measure the sleep/wake time differences on work-free days due to social jetlag and to calculate a corrected midpoint of sleep (MSF corrected) for both periods. Average sleep duration was calculated: five times the weekday sleep duration plus two times the weekend sleep duration divided by seven.

### Sleep phase delay

To assess the sleep phase delay, we subtracted the prior clock times from the clock times during the COVID-19 restriction phase. This resulted in four clock time differences, which were subjected to a factor analysis (principal component). All loaded onto the same single factor, labelled “delayed sleep phase” (58.9% of the variance explained). Week bedtime delay loaded with 0.833, weekend bedtime delay with 0.770, wake week delay with 0.757, and wake weekend delay with 0.705 onto the factor.

## Results

Bedtimes and wake times differed between prior to and during the COVID-19 restriction phase (see Table 1), with one exception: there were no significant differences between weekend bedtimes. The most striking difference occurred in wake times during the week. Participants got up approximately 1 hour later during the COVID-19 restriction phase, while bedtimes remained nearly stable. Sleep duration differed by about 22 min between the two time periods; thus, during the COVID-19 restriction phase, people slept on average nearly half an hour longer on weekdays, while there was no significant difference (about 6 min) on weekends. Average sleep duration was about 15 min longer. Midpoint of sleep (MS) on weekdays was about 45 min later, while midpoint of sleep on weekends was a few minutes earlier. When the correction algorithm was applied (MSFsc) to gain an unbiased chronotype measurement from clock times, we found no differences between prior to and during the COVID-19 restriction phase (*p* = 0.167). Social jetlag decreased from 1:39 ± 1:00 to 0:49 ± 0:42 min. The participants in our study slept longer and on weekdays later, while their social jetlag decreased and the corrected midpoint of sleep remained stable.

**Table 1:** Sleep parameters before and during the COVID-19 restriction phase

	During COVID-19 (M)	SD	Before COVID-19 (M)	SD	T	Df	*P-*value
Wake week	08:03	01:33	07:08	01:10	17.575	679	<0.001
Wake weekend	09:06	01:31	09:16	01:26	−4.633	676	<0.001
Bed week	23:52	01:30	23:19	01:11	13.072	679	<0.001
Bed weekend	00:27	01:33	00:31	01:28	−1.687	679	0.092
Sleep duration week	08:11	01:01	07:49	01:09	8.784	679	<0.001
Sleep duration weekend	08:39	01:05	08:45	01:12	−2.765	676	0.006
Sleep duration average	08:19	00:57	08:05	00:59	6.86	676	<0.001
MS weekday	03:57	01:26	03:13	01:02:04	17.295	679	<0.001
MS weekend	04:47	01:26	04:53	01:19:29	−3.482	676	0.001
MSFsc	04:36	01:29	04:33	01:20:21	1.385	676	0.167

*SD* standard deviation; *M* mean; *Df* degrees of freedom; *MS* midpoint of sleep; *MSFsc* midpoint of sleep corrected

We found differences in the correlations between sleep parameters and the MESSi prior to and during the COVID-19 restriction phase (see Table 2). Prior to the restriction phase MA had a positive relationship to sleep duration, while EV related negatively (not significant) to it. During the restriction phase sleep duration during the week and at the weekend was negatively correlated to the MA facet as well as the EV facet. The DI scale was positively correlated with both variables during the COVID-19 restriction phase and with the sleep duration on weekends beforehand. The relationships between midpoint of sleep on weekdays and weekends/average sleep duration/corrected midpoint of sleep as well as wake up and bedtimes prior to and during the COVID-19 restriction phase retained their direction of effect in almost all variables. Only the average sleep duration during the COVID-19 restriction phase was significantly negatively correlated with MA even though it showed a positive relationship (not significant) before the restrictions.

**Table 2:** Correlational analysis of sleep parameters before and during the COVID-19 restriction phase and the MESSi

	MA	EV	DI
Sleep duration week COVID-19	−0.099*	−0.106**	0.153**
Sleep duration week before	0.088*	−0.226**	0.054
Sleep duration weekend COVID-19	−0.107**	−0.095*	0.095*
Sleep duration weekend before	−0.113**	−0.045	0.157**
MS weekday COVID-19	−0.519**	0.574**	0.125**
MS weekday before	−0.327**	0.421**	0.007
MS weekend COVID-19	−0.542**	0.575**	0.102**
MS weekend before	−0.512**	0.560**	0.092*
Sleep duration average COVID-19	−0.111**	−0.113**	0.149**
Sleep duration average before	0.033	−0.201**	0.097*
MSFsc COVID-19	−0.520**	0.554**	0.111**
MSFsc before	−0.443**	0.499**	0.057
Wake week COVID-19	−0.514**	0.497**	0.166**
Wake week before	−0.243**	0.259**	0.032
Wake weekend COVID-19	−0.550**	0.509**	0.130**
Wake weekend before	−0.519**	0.497**	0.151**
Bed week COVID-19	−0.463**	0.585**	0.067
Bed week before	−0.328**	0.477**	−0.020
Bed weekend COVID-19	−0.465**	0.566**	0.061
Bed weekend before	−0.414**	0.523**	0.018

*MA* morning affect; *EV* eveningness; *DI* distinctness; *MS* midpoint of sleep; *MSFsc* midpoint of sleep corrected

*p < 0.05; **p < 0.01

Concerning the sleep phase delay, which is a compound measure of bedtimes’ and wake times’ delay on weekdays and at weekends, we found a significant influence of MA/EV and children living in the household (Table 3). There were no effects of gender, age, and occupation. Thus, the sleep phase delay affected all participants similarly and moved the sleep phase to later clock times.

**Table 3:** Predictor variables of sleep phase delay. Univariate general linear model with sleep phase delay (compound measure, see text)

Source of variation	Df	RMS	F	Sig	Partial eta-squared
Corrected model	11	7.739	8.718	<0.001	0.125
Constant	1	0.102	0.115	0.734	0.000
Age	1	0.002	0.003	0.958	0.000
MA	1	12.441	14.015	<0.001	0.021
EV	1	10.911	12.292	<0.001	0.018
DI	1	1.219	1.373	0.242	0.002
Sex	1	0.085	0.095	0.758	0.000
Profession	1	0.126	0.141	0.707	0.000
No. children	1	6.487	7.307	0.007	0.011
Sex * profession	1	0.001	0.001	0.970	0.000
Sex * no. children	1	1.450	1.634	0.202	0.002
Profession * no. children	1	0.049	0.055	0.814	0.000
Sex * profession * no. children	1	0.388	0.437	0.509	0.001

*Df* degrees of freedom; *RMS* root mean square; *MA* morning affect; *EV* eveningness; *DI* distinctness

**Fig. 1:**
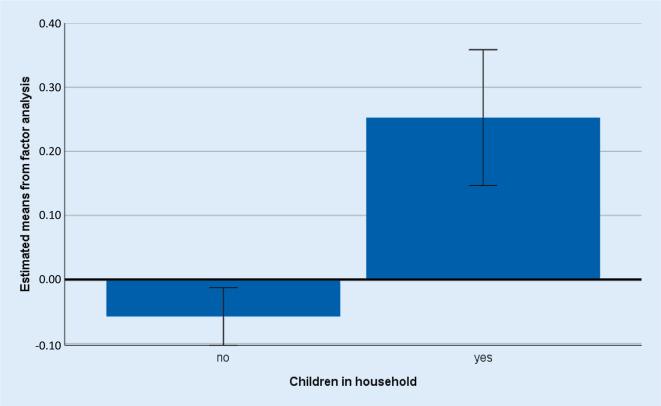
Sleep phase change in participants with and without children in the household. Estimated marginal means derived from the factor analysis

Regarding the number of children in households, we found a significant sleep phase shift in participants living with children (Table 3; Fig. 1), who slept longer and in whom sleep onset was later. Expectedly, MA was negatively (*r* = −0.285, *p* < 0.001) and EV positively (*r* = 0.266, *p* < 0.001) related to the sleep phase delay. Thus, circadian preference was a predictor of the sleep phase delay.

## Discussion

Participants working in home office slept longer and later during the COVID-19 restriction phase than before. The working situation participants were faced with compares partly to a modern approach to ways of working called “new ways of working” (NWW) [26]. Implementing NWW has the goal of creating temporal and spatial flexibility and thus accomplishing building work environments that focus on innovation and productivity while reducing costs [27]. The work situation in our sample was spatially flexible for every participant and temporally flexible for the majority. For those who had a certain temporal constraint, the omission of commuting times resulted in an additive time scope. NWW is proposed as a way to improve work time control and therefore allow employees to adjust their work to their private life [28] and biological needs (e.g., chronotype [13]). Our results are in line with these hypotheses. The significant changes in sleep–wake schedules and sleep duration after a prolonged phase of home office can be interpreted as an approach to participants’ own intrinsic sleep–wake rhythm. Results of morningness–eveningness’s relation to sleep phase delay confirm again that evening-oriented people benefitted from remote working during the COVID-19 restriction phase. These participants were able to adapt their sleep–wake cycle to their own internal clock rather than to work start times. In morning–oriented people, this delay is inevitably shorter because the work start times fit their biological clocks better. Nevertheless, most participants benefited from the changed working situation in terms of sleep health. Gao and Scullin [9] showed comparable results in a US sample. Leone et al. [12] published the effects on social jetlag in the only other study so far. The reported results correspond to ours.

Since schools in Germany implemented online teaching instead of attendance classes, students had a prolonged time scope in the morning lacking commuting times. Additionally, many schools started online teaching later. Therefore, students had to get up later in the morning, similar to their parents’/caregivers’ working situation. When controlling for the number of children in participants’ households, sleep phase delay increased. Our results show clearly that the negative effects of early school start times impact parents and caregivers as well as children. In general, the obtained results were comparable to other studies. Sinha et al. [10] showed that sleep onset and wake-up times were significantly delayed, with an average delay of sleep onset by 38 min and wake-up time by 51 min, irrespective of age and gender in an Indian sample [10]. Chinese and Italian people slept later and longer than usual during the COVID-19 quarantine phase [7, 11]. In another Italian sample similar results were found, but the impact of the delay in bedtime and in wake-up time was more pronounced in students [2]. These authors used a comparably structured sample with students and university staff. However, in our study, we found no differences between the groups (student versus non-student), despite a slightly higher sample size.

Furthermore, we found changes in the midpoint of sleep during the week as well as the weekend when comparing prior to and during the COVID-19 restriction phase. Only Leone et al. [12] have discussed this aspect in an Argentinian sample so far. One of the most intriguing results concerning the clock-based chronotype (midpoint of sleep corrected) was that the measurement method was not sensitive to sleep time changes during the restriction phase. This suggests that smaller changes in sleep–wake schedules do not necessarily reflect a general change in chronotype as a clock-based measure. However, Leone et al. [12] reported a shift toward a later midpoint of sleep (corrected) in their Argentinian sample. In addition, the circadian preference as measured by the MEQ [12] did not change between the two periods. This supports our findings that daytime preference, as well as the clock time-based measured chronotype, is stable. Otherwise, the validity of the clock time-based chronotype should be questioned. However, our data clearly support the corrected midpoint of sleep as a stable measure. Furthermore, the scales of the MESSi loaded onto the clock times as expected, both prior to and during the pandemic with a similar strength (Table 2). This provides additional validity for this newly developed measure.

## Limitations and strengths

One limitation of this study is the retrospective design. While the data on sleep–wake behaviour during the COVID-19 restriction phase were collected simultaneously, the data concerning the prior period were collected in retrospect. Furthermore, due to focusing on alterations following a change to home office, data might be biased. The recruitment might be limiting, since people who felt a change in sleep timing due to the altered work setting might be more interested in participating in the study than others. Another limiting factor is the high number of students taking part in the survey. Even though students’ learning environment changed to a home office situation too (e.g., classes were held online), the transferability to working in home office may not be given. In addition, the sample was non-representative (more than half of the participants had an academic background being students) and relatively unbalanced, with about twice as many females as males.

## Conclusion

In this study sample, sleep duration and sleep timing improved during the COVID-19 restriction phase. In addition, social jetlag regressed, which is beneficial to a healthy sleep and overall health. We could show that sleep duration during the week in evening-oriented participants in fact increased, while it decreased in morning-oriented participants. Overall, the sleep parameters changed positively in this sample. This study again shows that the social pressure following strict working hours is not target oriented in terms of health for a significant part of the population.
